# Clinical Outcomes Following Toric Intraocular Lens Implantation: A Case Series Study

**DOI:** 10.3390/jcm14072316

**Published:** 2025-03-28

**Authors:** Arie Y. Nemet, Olga Reitblat, Adi Levy, Achia Nemet, Ehud I. Assia

**Affiliations:** 1 Ein-Tal Eye Center, 15 Habrzel St., Tel Aviv 6971021, Israel; olga.reitblat@gmail.com (O.R.); adil@eintal.com (A.L.); assia@eintal.com (E.I.A.); 2 Department of Ophthalmology, Meir Medical Center, 59 Tshernichovsky St., Kfar Saba 4428164, Israel; 3 Department of Ophthalmology, Rabin Medical Center, Petach Tikva 4941492, Israel; 4 Department of Ophthalmology, Assuta Ashdod University Medical Center, Ashdod 7747629, Israel; achiant@gmail.com; 5 Sackler School of Medicine, Tel Aviv University, Tel Aviv 6997801, Israel

**Keywords:** cataract surgery, toric intraocular lenses, astigmatism, rotational stability

## Abstract

**Purpose:** This study aimed to assess the efficacy of for PODEYE TORIC intraocular lenses (IOL). **Methods:** This study was a retrospective, non-randomized, interventional case series. Inclusion criteria comprised diagnosis of an age-related cataract and a corneal astigmatism equal to or higher than 0.9 D and undergoing implantation of toric IOLs (TIOL). A single toric lens model (PodEye Toric, BVI) was used in all cases. **Results:** The study includes 51 eyes of 35 patients with TIOL implantation with a mean follow-up time of 45.7 (±36.5) days. Fourteen patients were targeted for mono-vision. Eight eyes had previously undergone refractive surgery (five post Myopic Lasik/PRK, two post RK/AK and one post RK). The mean postoperative adjusted spherical equivalent (SEQ) was −0.57 D ± 0.31 and the residual postoperative refractive astigmatism was −0.49 D ± 0.50. Only 2% of patients had a preoperative subjective astigmatism lower than 1.0 D whereas postoperatively, 94% of the patients had a residual astigmatism of 1.0 D or lower. The average deviation from the planned axis was 2.66 ± 0.26 degrees. None of the IOLs rotated to 10° or higher and 88% remained at 5° or less on the intended IOL axis. Twenty-six (63%) of IOL rotations were counterclockwise. **Conclusions:** PODEYE TORIC intraocular lenses provide exceptional refractive precision, reliable rotational stability, and consistently strong postoperative vision outcomes.

## 1. Introduction

Recent advancements in intraocular lenses (IOLs) have enhanced the accuracy and reliability of postoperative vision outcomes. Cataract removal surgery is a procedure for improving vision. Lately, toric IOLs have gained popularity in cataract removal for addressing astigmatism in the cornea. Despite the presence of astigmatism, it is possible to achieve satisfactory vision outcomes with toric IOLs [1,2]. The ability of a TIOL to maintain its position and orientation is essential for effective astigmatism correction. A slight shift in the orientation of a TIOL can diminish its ability to correct astigmatism by about 3.3% [3]. As the power of the IOL’s cylinder increases, the need for precise alignment and stability becomes more critical. Precise positioning of the IOL intraoperatively is essential for the long-term astigmatism control. An IOL rotation of less than 5° is usually satisfactory even for high-power cylinder correction. Preoperative corneal marking on the slit lamp of the desired final lens position is probably the most accurate method, but it is time consuming and requires a special setup of operating room routines. We usually reserve this methodology for high-power astigmatism, in addition to intraoperative axis marking [4]. Complications related to the rotational stability of IOLs have been reported years after the introduction of new toric IOLs, even for lenses that were very similar to those with established rotational stability [5,6]. This issue has been observed even with lenses that were not the same as existing TIOLs with proven rotational stability [7]. The POD platform is engineered with a distinctive double C-loop haptics design for superior fixation within the capsular bag, featuring a larger contact angle and four-point contact compared to standard IOLs. This platform has been demonstrated to accomplish the following:

- Allow for the even distribution of the compression forces at the haptic-capsular bag junction [8].
- Ensure the uniform spread of the squeezing pressures at the intersection point between the haptic-capsular bag [8].
- Keep the angle and vertical movement minimal [9].
- Achieve precise centering and stability in rotation [9].
- Moreover, this design facilitates effortless handling during the surgery by offering the choice to either rotate in a clockwise or counterclockwise direction, thereby decreasing the chance of improper alignment. The distinctive RidgeTech technology minimizes the occurrence of adhesive haptics on the lenses during and following the procedure.
- The aim of this research was to examine the accuracy of postoperative vision by measuring the error in predicting the residual astigmatism after the implantation of PODEYE, a monofocal toric intraocular lens (TIOL) (BVI) Physiol., (BVI).

## 2. Methods

### 2.1. Patients and Methods

This study is a retrospective, non-randomized interventional case series. The inclusion criteria included a diagnosis of an age-related cataract and a corneal astigmatism of 0.9 diopters or greater.

The study excluded patients with significant eye conditions that could affect surgery or its results, including problems with the cornea or retina (such as macular issues related to diabetes, myopia, or age-related degeneration).

The research team reviewed medical files of patients who underwent toric intraocular lens implants during cataract surgery, with all procedures performed by one surgeon (EIA). The research followed Helsinki Declaration guidelines and relevant medical protocols. The study received approval from Meir Medical Center’s Ethics Committee, which also waived the need for patient consent.

### 2.2. Clinical Protocol

“Clinical experience has shown that multiple measurements using various devices may significantly improve the precision of the IOL power as well as astigmatic correction. We therefore perform at least a double check using two biometric devices and two corneal topographers”. Each patient underwent pre-surgery measurements preoperatively, using four different devices: the Lenstar LS 900, IOLMaster 700, Pentacam tomographer, and Atlas topographer. All biometric readings met the stringent validation standards established by Warren Hill. (Warren Hill, East Valley Ophthalmology, https://doctor-hill.com/iol-power-calculations/validation-guidelines/, accessed on 25 November 2023). The surgeon chose the implanted TIOL using the Barrett online toric calculator with the predicted posterior astigmatism function (https://ascrs.org/barrett-toric-calculator, accessed on 25 November 2023) and the IOL manufacturers’ online calculators, (https://www.acrysoftoriccalculator.com/, https://www.physioltoric.eu/, https://www.Tecnistoriccalc.com/, accessed on 25 November 2023). The TIOL power and placement axis were calculated using the Lenstar measurement when agreement between all devices was achieved. When no agreement was achieved between Lenstar and the IOL Master, the measurements were repeated and occasionally a third biometer was used (Tomey 2000 OA, Tomey GmbH, Nagoya, Japan). The biometer data were compared to the visual and numerical data of the topographers and finally the measurements that were closer to the axis of the topographer and the tomographer were chosen for the IOL calculation. Cylinder correction was calculated using several formulas and averaged. In cases of dry eyes and irregular astigmatism, the patients were treated with lubricants for at least two weeks and the measurements were repeated. When no agreement was achieved between all devices, a toric IOL was not implanted.

### 2.3. Intraocular Lens

PODEYE TORIC is a single-piece Biconvex Aspheric Toric Monofocal IOL made of a hydrophobic acrylate.

The refractive index is 1.53, the total diameter of the IOL is 11.40 mm, and the optical zone diameter is 6.0 mm. The IOL consists of double C-loop haptics to reduce rotation of the lens and the cylindrical power at the IOL plane is available from +6.00 to +30.00 D. The manufacturer-labeled A-constant is 119.40.

### 2.4. Surgical Technique

The surgery procedures were all conducted at the Ein-Tal Eye Center in Tel-Aviv by one experienced surgeon (EIA). Local anesthetic drops were used for all patients. The Catalys femtosecond laser system (J&J) created toric marks on the cornea after marking the horizontal axis (0–180°) with the patient sitting upright. For patients with an astigmatism of 2.5 diopters or more, the surgical team made additional marks at the edge of the cornea using Haag-Streit slit lamps (Kniz, Switzerland) before the operation.

The surgical technique involved making a small, clear cut in the cornea (2.2–2.4 mm) and using phacoemulsification. After removing the cataract, the surgeon placed the intraocular lens into the capsular bag using an anterior chamber maintainer (ACM). This procedure avoided the need for an ophthalmic viscoelastic device (OVD), eliminating the step of removing the leftover OVD before the final positioning of the toric intraocular lens. The surgeon then rotated the lens to the intended angle and carefully pressed it back against the posterior capsule.

For data analysis, we evaluated and compared the effects of the IOL (cylinder and spherical power), corneal astigmatism (direction and power), axial length, and anterior chamber depth on the IOL deviation and its direction. TIOL deviation was defined as the difference between the planned axis of the TIOL and the actual alignment axis measured at least one month after surgery.

### 2.5. Statistical Analysis

Statistical analyses were performed using the R software version 4.2.0 (A Language and Environment for Statistical Computing, https://www.Rproject.org/, accessed on 27 December 2023). Descriptive statistics were presented as a mean ± standard deviation (SD) [range], and categorical values were summarized with their relative percentage.

The prediction error (PE) for the Barrett Universal II formula was calculated by subtracting the predicted refraction from the postoperative refraction spherical equivalent (SEQ), adjusted for a refraction examination lane of 6 m, for the IOL that had been implanted. The prediction error of the refractive astigmatism was calculated as the difference between the refractive astigmatism and the residual astigmatism as predicted by the Barrett Toric Calculator. The Eyetemis Analysis Tool (available at: www.eyetemis.com, accessed on 27 December 2023) was used for the analyses of (1) Trueness, calculated as the distance of the PE to zero; (2) Precision, calculated as the distance between the PE and the mean PE; and (3) Accuracy, calculated as the absolute PE (developed by Adi Abulafia MD, PhD, Yuval Benjamini, PhD, and Yoav Kan-Tor, PhD available at: www.eyetemis.com, accessed on 27 December 2023). Data analysis was performed for the entire cohort and separately for regular eyes and eyes that had undergone previous refractive surgery.

## 3. Results

The study includes 51 eyes from 35 patients who underwent toric intraocular lens (TIOL) implantation, with a mean follow-up time of 45.69 (±36.58) days. Fourteen patients were targeted for mono-vision. Eight eyes had undergone refractive surgery (five post Myopic Lasik/PRK, two post RK/AK, and one post RK). The mean post-op adjusted SEQ was −0.57 D ± 0.31 and the postoperative refractive astigmatism was −0.49 D ± 0.50. The patient’s demographics, preoperative biometric data, IOL characteristics and postoperative data for the entire cohort and by groups are summarize in Table 1. None of the difference between the two groups was statistically significant.

The average deviation from the planned axis was 2.66 ± 0.26 degrees. Twenty-six had a counterclockwise (CCW) rotation and fifteen had a clockwise (CW) rotation. The entire distribution of frequencies is shown in Table 2.

**Figure 1:**
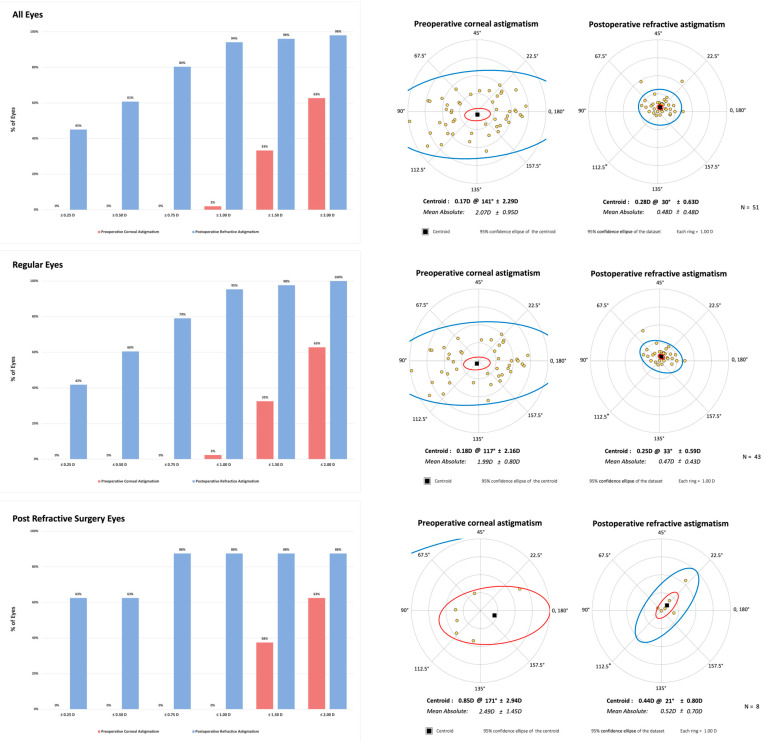
The preoperative corneal astigmatism and the postoperative refractive astigmatism in both absolute and centroid presentations.

Figure 1 shows the preoperative corneal astigmatism and the postoperative refractive astigmatism in both absolute and centroid presentations.

**Figure 2:**
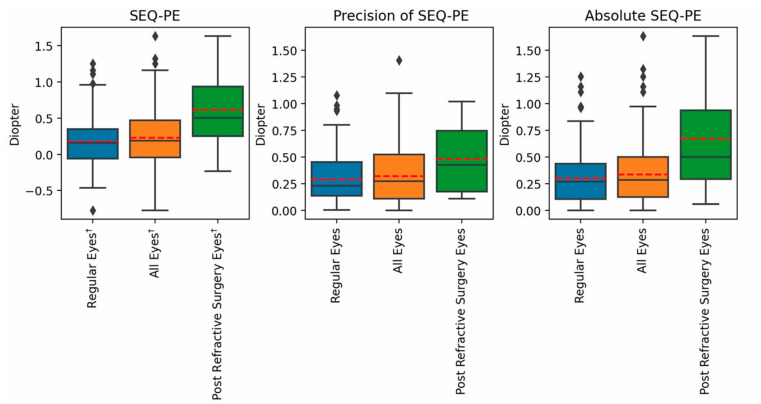
Box blots for the PE in the SEQ.

**Figure 3:**
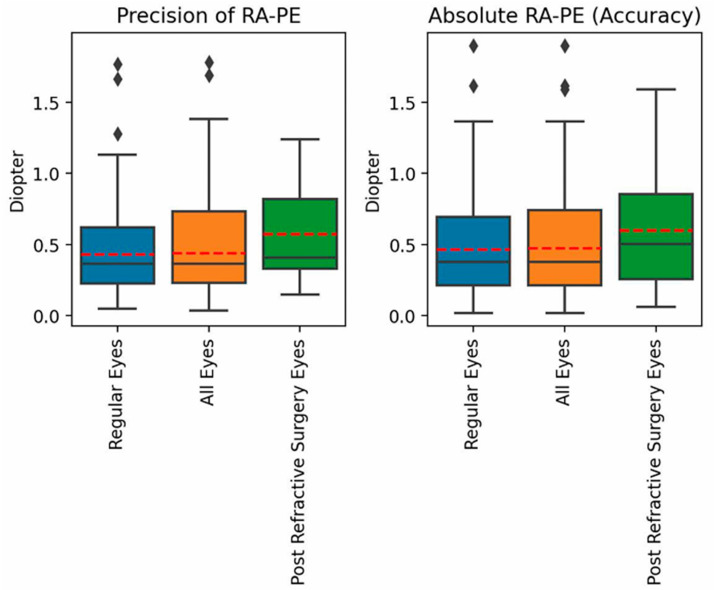
Box blots for the PE in refractive astigmatism.

Box blots for the PE in the SEQ are presented in Figure 2. Notably, a hyperopic error was observed for both groups. Box blots for the PEs in refractive astigmatism are presented in Figure 3. Across all Precision and Accuracy indices for the PEs in both SEQ and refractive astigmatism, regular eyes showed a better trend in comparison to post refractive eyes.

Complete data summaries for the Trueness, Precision, and Accuracy metrics of the PE in the SEQ and refractive astigmatism are presented in Supplementary Table S1. The cumulative percentages of eyes with an SEQ PE and astigmatic PE within ±0.25, ±0.50, ±0.75 and ±1.00 D are presented in Supplementary Figures S1 and S2, respectively.

## 4. Discussion

This study evaluated the visual outcomes and rotational stability of a toric intraocular lens (TIOL) platform. Our findings demonstrated high predictability and a low incidence of deviations from the intended axis. Toric IOLs, initially introduced by Shimizu et al. in 1992, were designed as three-piece, non-foldable polymethyl methacrylate implants to be inserted through a 5.7 mm incision [10]. A preoperative corneal astigmatism of 1 diopter (D) or greater is observed in over one-third of patients undergoing cataract surgery, with 22% exhibiting more than 1.5 D and 8% presenting with more than 2.0 D of astigmatism. In these cases, the use of TIOLs is instrumental in achieving postoperative spectacle independence and ensuring optimal patient satisfaction [11,12,13].

### Rotational Stability of Toric IOL

Postoperative toric intraocular lens (TIOL) misalignment is a significant factor contributing to suboptimal visual outcomes following toric IOL implantation. Effective correction of astigmatism with a TIOL necessitates precise measurements and calculations of power, the corneal astigmatism axis, accurate intraoperative marking and alignment of the TIOL, and robust postoperative rotational stability [4,14].

The impact of IOL decentration and tilt on visual function varies based on the IOL design [15]. For toric IOLs, decentration and tilt can lead to less predictable astigmatism correction [16,17]. The haptic design and overall IOL diameter may play a role in stabilizing the IOL [18]. Previous studies have shown that unsatisfactory IOL rotational stability can emerge years after the introduction of new toric IOLs [6,18]. However, recent findings by Inoue et al. indicate that nearly all TIOL axis rotation (mean of 4.09 degrees) occurs within the first hour post-surgery [19]. Lee and their research team found that any rotation of the lens happens in the early period after surgery. They reached this conclusion because patients checked either one hour after surgery or the next day showed the same alignment results [20]. Newer toric IOLs have shown enhanced rotational stability, providing precise visual outcomes with minimal higher-order aberrations [14]. The toric lenses were calculated to correct the full amount of astigmatism, preferably leaving a small amount (<0.25 D) of with-the-rule astigmatism to compensate for the natural against-the-rule deviation with age. An average of 9% deviation from the calculated cylinder is, in most cases, negligible and does not require surgical intervention. A 2.6° deviation is so small that it may occur even in eyes with perfect marking and no postoperative rotation.

The more common counterclockwise rotation is probably characteristic to the specific design of the PDYEYE lens. The “double-mustache” shape is symmetrical and therefore the ease in, or resistance to, rotation is similar in the two directions. This is in contrast to the double C-loop design of most posterior chamber IOLs that would allow an easier rotation in the clockwise direction.

Patients should be clearly instructed postoperatively not to move their head aggressively in the first couple of days, and especially in the first couple of hours. This, combined with modifications in haptic designs and configurations, significantly reduced, and almost eliminated, a large rotation of toric lenses located in the capsular bags

Realignment of the TIOL is necessary in cases of clinically significant residual astigmatism, typically following more than 10° of rotation from the target axis [14]. A rotation of less than 0.5 D usually does not require additional intervention [21]. A 10° rotation can result in a loss of one-third of the cylindrical effect, e.g., 0.5 D in an eye with 1.5 D of astigmatism, but 1.5 D in an eye with 4.5 D of astigmatism. Realignment may be required in 0.65–3.3% of cases, with an incidence of 1.1% observed with AcrySof toric IOLs, 2.3% with older versions of the Tecnis Toric IOL, and 3.3% with silicone lenses [22,23,24].

In the current study, we observed an average IOL rotation of 2.66° (a 9% reduction in cylindrical effect) in eyes with a mean corneal astigmatism of 2.0–2.5 D, resulting in negligible residual effect in most cases. The investigated IOL demonstrated an excellent low rate of deviation from the planned axis, with a counterclockwise (CCW) rotation found in 63.4%. Sheen et al. found similar deviations without a predominant direction [4].

Draschl et al. evaluated the rotational stability of nontoric IOLs of the same design but different materials (Pod Ay 26P and Pod Eye), showing that the hydrophobic IOL (1.6 ± 1.61°) was more rotationally stable than the hydrophilic IOL (2.4 ± 1.85°) while both provided good capsular bag stability [9]. Vandekerckhove demonstrated that trifocal toric IOLs offer better rotational stability than monofocal IOLs, likely due to the higher frictional coefficient of their surface [7]. Ribeiro et al. assessed the efficacy of five calculators for toric IOLs in cataract patients undergoing trifocal toric IOL implantation, finding that the PhysIOL and Barrett toric calculators, which account for posterior corneal astigmatism, yielded lower astigmatic prediction errors compared to standard calculators based on anterior keratometry data only [2].

Studies have reported a range of TIOL misalignments, with most showing less than 5 degrees for both AcrySof and Tecnis TIOLs [11,24]. One study of 30 Tecnis TIOL eyes reported a mean misalignment of 7.7 degrees [25], and Inoue reported 6.67 degrees with the Tecnis [19]. Exceptions include the Lentis L-312T, which was withdrawn from the market due to a mean misalignment of 20 degrees [6]. Lee et al. showed that the AcrySof TIOL had significantly greater rotational stability (91.9% of eyes rotated ≤ 5 degrees) compared to the Tecnis TIOL (81.8%) [20]. Sheen-Ophir et al. recently found similar low deviations from the planned axis among three common TIOLs with no significant difference in the average deviation or its direction [4].

Osawa et al. [26] compared four models of toric IOLs, including AcrySof, TECNIS, HOYA 355, and HOYA XY-1, finding no significant differences in the amount of IOL tilt (approximately 5°) and decentration (0.2 mm) among the models. Modifications to these lenses, such as increased overall length, faster unfolding, and textured haptic surfaces, have significantly improved postoperative rotational stability [26].

Manual marking accuracy has been validated by several studies, with the three-step marking method demonstrating a total error of 4.9 ± 2.1° in toric IOL alignment. In this study, we used the POD IOL, a 1-piece IOL with two symmetrical bifid open-loop haptics and an overall diameter of 11.4 mm, which is smaller than other lenses. The unique symmetrical configuration of this platform allows or easy rotation in both directions during surgery. Both the AcrySof SN6AT and Symfony ZXT lenses measure 13.0 mm across and feature C-loop haptics. While the POD FT TIOL has a smaller overall diameter, its distinctive double C-loop haptic design may provide equivalent stability to compensate for its more compact size [27].

Additionally, the mechanical properties of the haptic material, including its memory and adherence to the capsular bag, influence rotational stability. Patel et al. found that postoperative rotation was significantly higher with loop-haptic IOLs than with plate-haptic IOLs [28]. Clinical results in eyes following refractive procedures were similar to those in regular eyes, despite being generally longer, more myopic, and having more astigmatism. These corneas were flatter and thinner, but other parameters such as white-to-white (WTW), lens thickness (LT), and anterior chamber depth (ACD) were similar to those of regular eyes, indicating no need for adjustments in toric power or positioning.

The limitations of this study include its retrospective design and lack of randomization. Another limitation is the relatively short-term follow-up of the study. Even though most IOL cylindrical correction and axis rotation occurs within days, it is essential to further investigate the long-term stability of toric lenses. Additionally, current TIOL axis measurement techniques are not free of error.

## 5. Conclusions

Our study evaluated the TIOL axis position at least one month postoperatively, based on the assumption that no further deviation would occur beyond this period. The results indicate that the PODEYE TORIC models exhibit only small and consistent deviations from the planned axis. These findings align with most studies, which report misalignments of 5 degrees or less [4,11,20,24,26,29]. Eye characteristics such as axial length, anterior chamber depth, and the direction of corneal astigmatism were found to have minimal influence on the degree of deviation. Consequently, toric IOLs can and should be used to correct clinically significant corneal astigmatism. Using modern devices to measure and calculate IOL power and the desired axis and using modern lenses with improved haptic designs, such as the IOL in this study, may further improve the clinical results and spectacle independence.

## Figures and Tables

**Table 1:** Baseline characteristics.

Parameter	All Eyes	Regular Eyes	Post Refractive Surgery Eyes
Eyes	51	43	8
Gender F;M	30 (58.8%); 21 (41.2%)	22 (51.2%); 21 (48.8%)	8 (100%); 0(%)
Age (years)	71.31 (9.96)	71.77 (10.28)	68.88 (8.17)
Laterality (RE)	29 (56.9%)	24 (55.8%)	5 (62.5%)
Distance; Monovision	37 (72.5%); 14 (27.5%)	31 (72.1%); 12 (27.9%)	6 (75%); 2 (25%)
Axial Length (mm)	24.68 (1.6)	24.41 (1.52)	26.08 (1.29)
Average K (D)	43.51 (2.19)	44.03 (1.67)	40.71 (2.65)
Preoperative Corneal Astigmatism (absolute mean) (D)	2.07 (0.96)	1.99 (0.81)	2.49 (1.55)
Preoperative Corneal Astigmatism (centroid mean) (D)	0.17 (2.29) 141°	0.18 (2.16) 117°	0.85 (2.94) 171°
IOL Power (D)	19.38 (5.12)	19.34 (5)	19.62 (6.09)
IOL Cylinder (D)	2.81 (1.23)	2.77 (1.02)	3.06 (2.12)
Postoperative Refraction SE (D)	−0.57 (0.81)	−0.58 (0.86)	−0.53 (0.47)
Postoperative Refraction Cylinder (absolute mean) (D)	−0.48 (0.48)	−0.47 (0.43)	−0.52 (0.70)
Postoperative Refraction Cylinder (centroid mean) (D)	0.28 (0.63) 30°	0.25 (0.59) 33°	0.44 (0.8) 21°

Values are mean (SD) or n (%).

**Table 2:** Toric IOL rotation summary.

Parameter	All Eyes (n = 47)	Regular Eyes (n = 39)	Post Refractive Surgery Eyes (n = 8)
Mean (SD) [range] Rotation Direction	2.66 (2.26) [0–9]	2.59 (2.22) [0–9]	3 (2.56) [0–7]
Rotation Direction	CCW: 26 (63.4%); CW: 15 (36.6%)	CCW: 24 (68.6%); CW: 11 (31.4%)	CCW: 2 (33.3%); CW: 4 (66.7%)
0° Rotation	6	4	2
1° Rotation	14	14	0
2° Rotation	7	5	2
3° Rotation	4	3	1
4° Rotation	7	6	1
5° Rotation	3	3	0
6° Rotation	3	2	1
7° Rotation	1	0	1
8° Rotation	1	1	0
9° Rotation	1	1	0

## Data Availability

The original contributions presented in the study are included in the article, further inquiries can be directed to the corresponding authors.

## Supplementary Materials

The following are available online at https://www.mdpi.com/article/10.3390/jcm14072316/s1, Figure S1: The cumulative percentage of eyes with a SEQ PE and astigmatic PE, within ±0.25, ±0.50, ±0.75 and ±1.00 D; Table S1: Trueness Precision, and Accuracy of the prediction error in the SEQ and refractive astigmatism. Figure S2: The cumulative percentage of eyes with an astigmatic PE within ±0.25, ±0.50. ±0.75 and ±1.00 D.
