# Supplementary material for: Clinical Outcomes Following Toric Intraocular Lens Implantation: A Case Series Study
Source: J Clin Med. 2025 Mar 28;14(7):2316. doi: 10.3390/jcm14072316 (PMC11989974; doi:10.3390/jcm14072316)
Supplement: Supplementary file 1 [file jcm-14-02316-s001.zip › jcm-3448309-supplementary.pdf]

Supplementary Materials:

Table S1. Trueness Precision, and Accuracy of the prediction error in the SEQ and refractive astigmatism.

statistic	group	count	mean	SD	min	25%	50%	75%	max	rms
SEQ-PE	All Eyes	51	0.27	0.49	-0.78	-0.04	0.19	0.47	1.64	0.56
SEQ-PE	Post Refractive Surgery Eyes	8	0.61	0.62	-0.24	0.25	0.50	0.94	1.64	0.85
SEQ-PE	Regular Eyes	43	0.20	0.44	-0.78	-0.06	0.16	0.35	1.26	0.48
Precision of SEQ-PE	All Eyes	51	0.37	0.33	0.00	0.11	0.27	0.52	1.41	0.49
Precision of SEQ-PE	Post Refractive Surgery Eyes	8	0.48	0.35	0.11	0.18	0.43	0.75	1.02	0.58
Precision of SEQ-PE	Regular Eyes	43	0.33	0.29	0.00	0.14	0.23	0.45	1.08	0.44
Absolute SEQ-PE	All Eyes	51	0.40	0.39	0.00	0.13	0.29	0.50	1.64	0.56
Absolute SEQ-PE	Post Refractive Surgery Eyes	8	0.67	0.55	0.06	0.30	0.50	0.94	1.64	0.85
Absolute SEQ-PE	Regular Eyes	43	0.35	0.34	0.00	0.11	0.27	0.44	1.26	0.48
Precision of RA-PE	All Eyes	51	0.50	0.40	0.03	0.23	0.36	0.73	1.78	0.64
Precision of RA-PE	Post Refractive Surgery Eyes	8	0.57	0.38	0.15	0.33	0.41	0.82	1.24	0.67
Precision of RA-PE	Regular Eyes	43	0.49	0.40	0.05	0.23	0.36	0.62	1.77	0.63
Absolute RA-PE (Accuracy)	All Eyes	51	0.54	0.43	0.02	0.21	0.38	0.74	1.90	0.69
Absolute RA-PE (Accuracy)	Post Refractive Surgery Eyes	8	0.60	0.51	0.06	0.26	0.50	0.86	1.59	0.77
Absolute RA-PE (Accuracy)	Regular Eyes	43	0.52	0.42	0.02	0.21	0.38	0.70	1.90	0.67

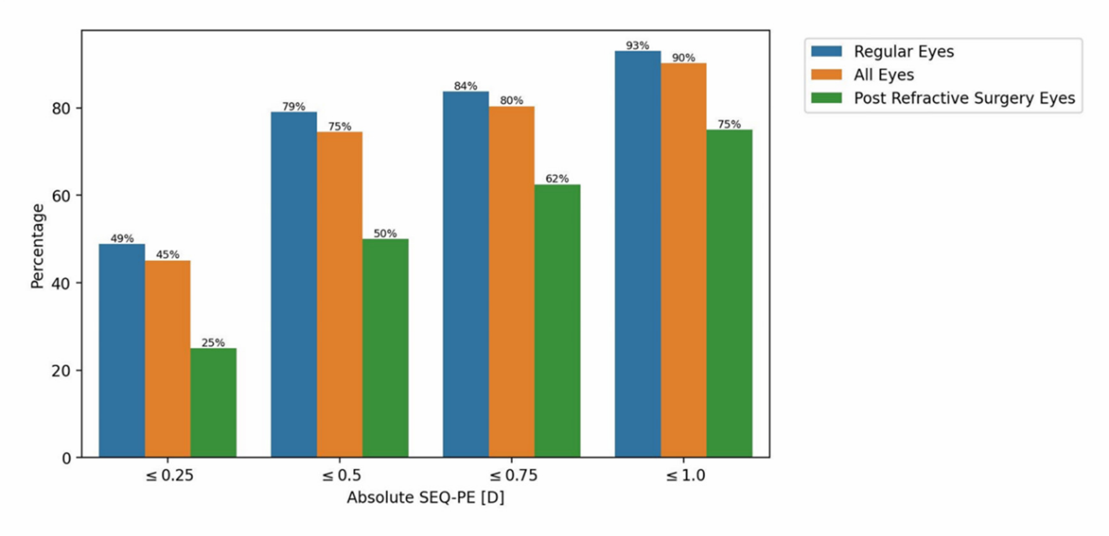

Figure S1. The cumulative percentage of eyes with a SEQ PE and astigmatic PE, within ± 0.25, ± 0.50. ± 0.75 and ± 1.00 D.

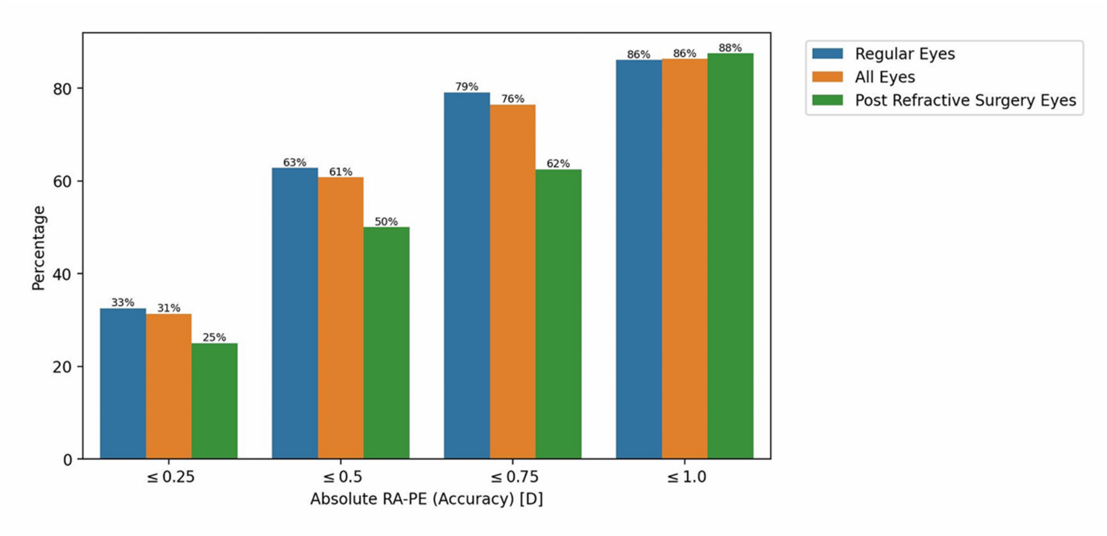

Figure S2. The cumulative percentage of eyes with an astigmatic PE within ±0.25, ±0.50. ±0.75 and ±1.00 D
